# Bioinformatic analysis of genotype by sequencing (GBS) data with NGSEP

**DOI:** 10.1186/s12864-016-2827-7

**Published:** 2016-08-31

**Authors:** Claudia Perea, Juan Fernando De La Hoz, Daniel Felipe Cruz, Juan David Lobaton, Paulo Izquierdo, Juan Camilo Quintero, Bodo Raatz, Jorge Duitama

**Affiliations:** 1 Agrobiodiversity Research Area, International Center for Tropical Agriculture (CIAT), Cali, 763537 Colombia; 2 Department of Plant Biotechnology and Bioinformatics, Ghent University, Ghent, 9052 Belgium; 3 Gerencia de Procesos, Centro Médico Imbanaco, Cali, 760033 Colombia

**Keywords:** Bioinformatics, GBS, Sequencing, SNP calling, NGSEP

## Abstract

**Background:**

Therecent development and availability of different genotype by sequencing (GBS) protocols provided a cost-effective approach to perform high-resolution genomic analysis of entire populations in different species. The central component of all these protocols is the digestion of the initial DNA with known restriction enzymes, to generate sequencing fragments at predictable and reproducible sites. This allows to genotype thousands of genetic markers on populations with hundreds of individuals. Because GBS protocols achieve parallel genotyping through high throughput sequencing (HTS), every GBS protocol must include a bioinformatics pipeline for analysis of HTS data. Our bioinformatics group recently developed the Next Generation Sequencing Eclipse Plugin (NGSEP) for accurate, efficient, and user-friendly analysis of HTS data.

**Results:**

Here we present the latest functionalities implemented in NGSEP in the context of the analysis of GBS data. We implemented a one step wizard to perform parallel read alignment, variants identification and genotyping from HTS reads sequenced from entire populations. We added different filters for variants, samples and genotype calls as well as calculation of summary statistics overall and per sample, and diversity statistics per site. NGSEP includes a module to translate genotype calls to some of the most widely used input formats for integration with several tools to perform downstream analyses such as population structure analysis, construction of genetic maps, genetic mapping of complex traits and phenotype prediction for genomic selection. We assessed the accuracy of NGSEP on two highly heterozygous F1 cassava populations and on an inbred common bean population, and we showed that NGSEP provides similar or better accuracy compared to other widely used software packages for variants detection such as GATK, Samtools and Tassel.

**Conclusions:**

NGSEP is a powerful, accurate and efficient bioinformatics software tool for analysis of HTS data, and also one of the best bioinformatic packages to facilitate the analysis and to maximize the genomic variability information that can be obtained from GBS experiments for population genomics.

**Electronic supplementary material:**

The online version of this article (doi:10.1186/s12864-016-2827-7) contains supplementary material, which is available to authorized users.

## Background

Genotype by sequencing (GBS) is a powerful, cost-effective method to obtain genome-wide variability information for populations composed by hundreds of individuals [1–5]. In brief, a GBS protocol starts with a digestion of the DNA using one or more known restriction enzymes, aiming to reduce the complexity of the genome to be sequenced. Then, fragments of suitable lengths (less than 800 bp) are ligated to adapters, amplified and sequenced in a high throughput Illumina platform [6]. Multiple samples can be sequenced in one single lane adding appropriate barcodes [7]. Sequenced reads can then be demultiplexed and either analyzed de-novo or aligned to a reference genome if available. In the latter case, variants can be identified using analysis pipelines similar to those used for analysis of whole genome resequencing data [8]. The main feature of this protocol is that a relatively small, yet reasonably well distributed and reproducible, portion of the entire genome is sequenced, which allows to identify and genotype thousands of genomic variants across the genome and across different samples. GBS is becoming the method of choice for several applications in plant genomics and plant breeding [9], such as the analysis of population dynamics [2], construction of high density genetic maps [4, 8], genetic mapping of complex traits through Genome-Wide Association Studies (GWAS) [3] and estimation of breeding values in genomic selection [1, 5].

A key component of any GBS protocol is the bioinformatics pipeline required to analyze the reads and to obtain polymorphic sites within the sequenced population. Widely used packages such as bwa [10] or bowtie2 [11] for short read alignment and Samtools [12] or GATK [13] for variants detection and genotyping have been used to analyze GBS data [8]. More recently, custom packages such as Stacks [14] or the Tassel GBS pipeline [15] have been developed specifically for analysis of the types of reads produced by GBS technologies. The main advantage of these methods over previous approaches is that they can still work in the absence of a reference genome [16]. Tassel in particular takes advantage of the nature of GBS reads to perform a highly efficient calculation of genomic variants.

We recently developed the software package NGSEP as an accurate, efficient and easy-to-use alternative for analysis of high throughput sequencing (HTS) data and we demonstrated its advantages over other commonly used packages using benchmark whole genome sequencing (WGS) data on humans, yeast and rice [17]. Here we describe the new functionalities implemented in NGSEP for analysis of GBS data and we compare the performance of NGSEP with other commonly used methods for variants detection and genotyping on three different breeding populations of cassava and beans.

## Results

### Novel functionalities implemented in NGSEP

#### Deconvolution

The deconvolution process allows to distribute the reads obtained from fragments barcoded and sequenced in one Illumina lane, producing one separate file for each sample. It receives a fastq file with the lane information and a text file describing the link between barcodes and samples. Although deconvolution can be generally applied to any type of sequencing in which samples are identified by barcodes, it is particularly important for GBS data because in GBS experiments 96 samples (or even up to 384 samples in some cases) are sequenced per lane to achieve cost efficiency. We also included an option in this module to identify a user-defined tag on each read and then trim the read from the starting point of this tag. This allows to remove contamination of adaptor sequence on the three prime end of the reads, which we have identified as the most important issue affecting the quality of the reads produced by the Elshire protocol for GBS (see “Methods” for details).

#### One step wizard for parallel multisample analysis

**Fig. 1:**
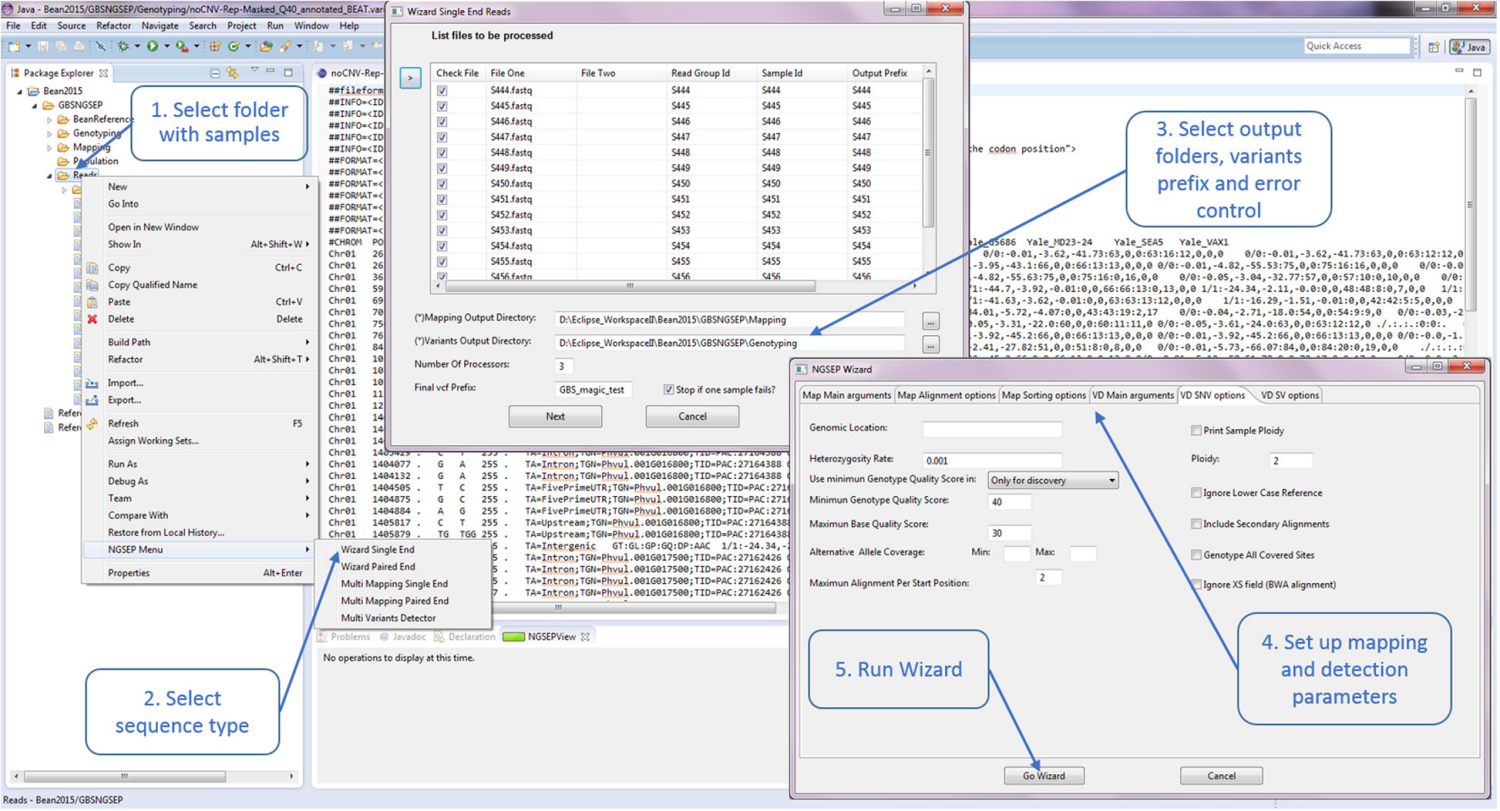
NGSEP wizard. One step wizard to obtain population variability datasets

The NGSEP Wizard is a new functionality which, starting from raw sequencing data, embodies all steps required to obtain a population variants file with only one execution screen. This functionality greatly reduces the amount of work needed by researchers to obtain variation datasets over populations and also helps to standardize parameters for read aligment and variants detection across different samples. Figure 1 shows an schematic of the user interaction needed to run this functionality. The wizard starts when users select a folder including raw sequencing data in fastq format for different samples. Users then select the samples to process and choose parameters and output directories for read alignment, variants identification and genotyping and finally submit the entire pipeline. For error control, the wizard either has the option of skipping samples with problems in any step and trying to finish the process including only the successful samples, or it can also cancel the entire execution in case that one sample fails. For both cases NGSEP will notify about the samples with problems both through execution logs and showing a popup alert summarizing errors across the different samples. Although the NGSEP wizard can in principle be applied to HTS reads obtained from any type of sequencing protocol, it is particularly useful to facilitate to users the process of obtaining variation datasets from raw reads in GBS experiments in which hundreds of samples are sequenced in just a few lanes and hence execution parameters need to be standardized across samples. This wizard covers a large part of the bioinformatic analysis steps included in currently used GBS protocols [7, 9].

#### Filtering and conversion of VCF files

We expanded the variants call format (VCF) filtering module to include most of the filtering strategies that can be employed to achieve the balance between variant genome coverage and genotype quality required for different applications. Genotype calls can be filtered based either on genotype quality (GQ field in the VCF genotype format) or total read coverage. Genomic regions can be either selected or filtered out, which is useful to remove markers in undesired regions (for example, repetitive elements) or to focus on a particular region of the genome, such as a quantitative trait locus (QTL) or a gene. Samples can also be either selected or filtered out, which allows users to remove samples that were sequenced at lower coverage or to focus on subpopulations within the whole dataset. Single nucleotide polymorfisms (SNPs) can then be selected based on several criteria such as number of individuals genotyped, minor allele frequency (MAF), distance between markers and functional annotations. To select SNPs ameanable for other high throughput SNP genotyping platforms (e.g. PCR based), users can also select limits on the GC-content of the region surrounding each particular SNP. All these filtering approaches can be applied to any VCF file using minimal computational resources, which facilitates users trying several different options for downstream analysis. We also expanded the conversion module to allow users to translate the genotype data to the input formats needed by tools such as DARwin to build distance-based dendograms [18], JoinMap for construction of genetic maps [19], and the R package rrBLUP to perform estimation and cross validation of breeding values in genomic selection [5].

#### Statistics on genotyped populations

We added three modules providing several statistics on the genotypes included in a VCF file. Summary statistics include counts of number of variants discriminated as biallelic SNPs, biallelic indels and other (multiallelic) variants, the MAF distribution and the distribution of SNPs genotyped in different numbers of individuals. It also includes counts of genotype calls per sample, including number of genotyped variants, non-reference genotype calls and heterozygous calls. Counts per sample are also discriminated by gene functional annotations. The diversity statistics module calculates for each variant common statistics for population genomic analysis such as MAF, expected and observed heterozygosity, and deviation from Hardy-Weinberg Equilibrium. Users can provide a text file indicating a clustering of samples in subpopulations, which allows this module to calculate diversity statistics for each subpopulation, facilitating the calculation and genome-wide analysis of F-statistics. Finally, a third function allows to compare the genotype calls on two VCF files (or one VCF file with itself), and calculates the number of differences between every pair of samples. This allows to identify potentially duplicated materials or direct parental relationships.

#### Imputation

One of the main issues of GBS, compared to high throughput genotyping platforms, is the relatively high percentage of missing data produced by the random distribution of read coverage both across samples and across genomic sites. Although several bioinformatics methods have been developed to perform genotype imputation (see [20, 21] for example), most of these tools have been developed in the context of imputation on human populations of unrelated individuals. Even though imputation on populations of inbred materials and biparental or multiparental populations is relatively easier because these populations generally have larger linkage disequilibrium (LD) blocks [22], very few bioinformatic tools are available to perform accurate genotype imputation on these cases. Keeping this in mind, we reimplemented the haploid version of the hidden markov model (HMM) implemented in fastPHASE [21] to perform imputation in populations of inbred lines, either unrelated or members of a breeding population. Our implementation receives and produces its output as a VCF file which allows users to directly integrate imputed genotypes in GWAS analysis using the format conversion module of NGSEP.

### Comparison of methods on cassava F1 breeding populations

**Fig. 2:**
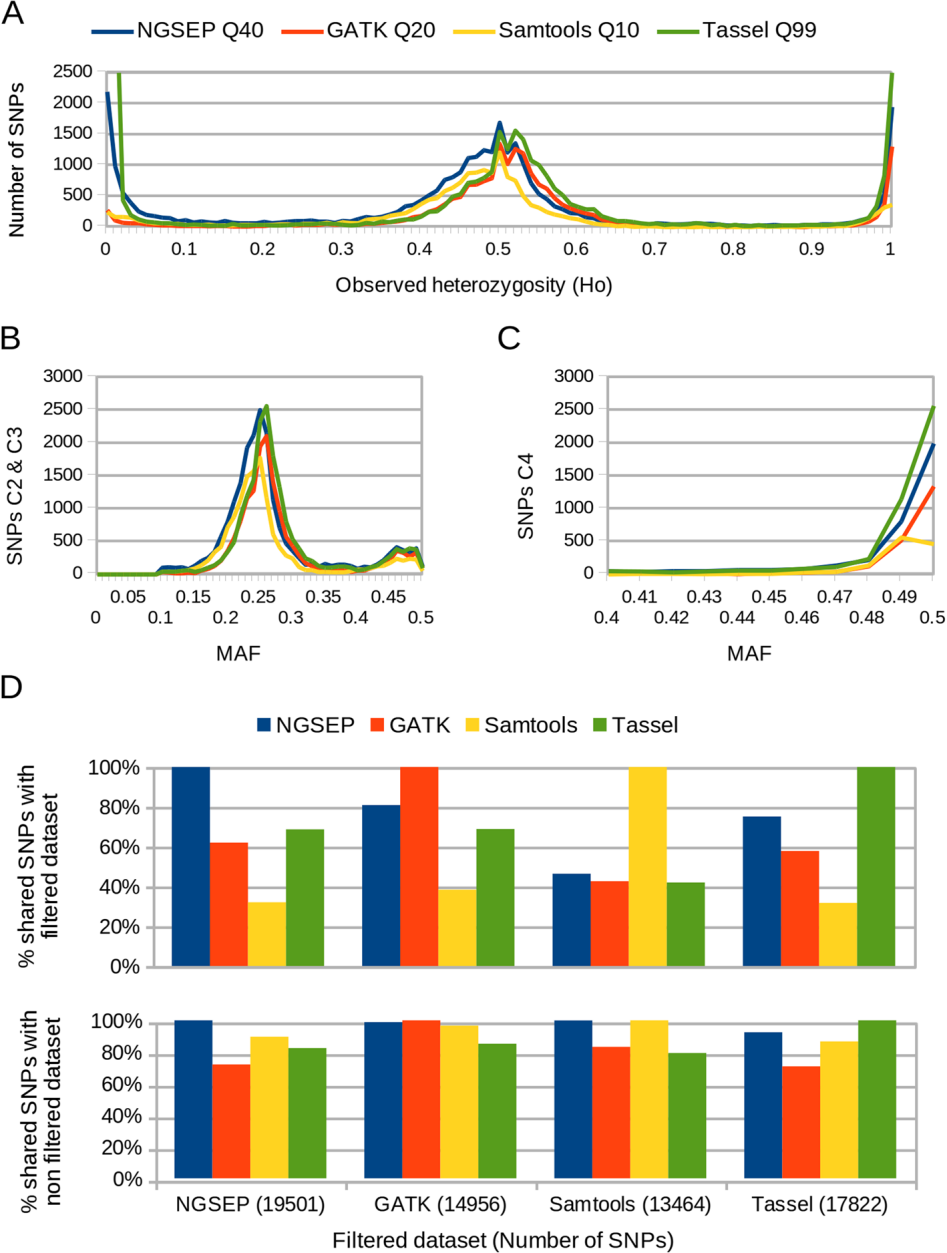
MAF and *H*_*o*_ distributions. Statistics on filtered SNPs obtained running the four discovery pipelines compared in this study on the K family GBS data. **a** Distribution of observed heterozygosity **b** MAF distribution in SNPs useful to build a genetic map (categories 2 and 3, see Methods for details), **c** MAF Distribution on highly heterozygous SNPs (category 4), and **d** Percentage of filtered SNPs useful to build a genetic map that appear at the filtered (upper chart), and unfiltered (lower chart) datasets obtained running each method

We evaluated the performance of NGSEP compared to some of the most widely used pipelines for SNP discovery and genotyping, namely GATK [13], Samtools [12] and Tassel [15], on GBS reads previously sequenced and used to generate a dense SNP based cassava genetic map [4, 8]. These reads were obtained from GBS experiments performed on two biparental full-sib cassava families of F1 crosses termed the K family [4] and the NxA family [8] (see “Methods” for details). Because cassava is a naturally outcrossing species, its cultivars hold high levels of heterozygosity. Hence, cassava F1 populations make a useful benchmark to assess the accuracy of different pipelines to identify both homozygous and heterozygous genotypes, using Mendelian rules of segregation as a gold-standard for expected genotype calls. We classified SNPs based on their observed heterozygosity (*H*_*o*_) and minor allele frequency (MAF) in four different categories (see Methods for details): 1) Monomorphic (AAxAA), 2) Homozygous/Heterozygous (AAxAa), 3) double heterozygous (AaxAa), and 4) segregating (AAxaa). Distributing the SNPs on these categories is useful to understand the behavior of each method, the amount and main sources of errors and also the consequences of these errors for downstream analysis. For each category we calculated as measures of sensitivity the number of genotype calls in categories 2, 3 and 4 and, as measures of specificity, the number of segregation errors and the number of SNPs in category 1. Figure 2 shows that the distribution of *H*_*o*_ and MAF on datasets with relatively equivalent quality obtained running the four pipelines is generally consistent with expected segregation patterns. This figure also suggests that all methods included in this comparison are able to provide thousands of SNP markers genotyped with high accuracy.

We compared the number of shared SNPs between the different methods after keeping genotype calls with comparable genotype quality (see next paragraphs for details), and applying the same filters on number of individuals genotyped, repetitive regions and observed heterozygosity, retaining SNPs consistent with the categories useful to build a genetic map (C2 and C3). We found that, among filtered datasets, NGSEP, GATK and Tassel share over 60 % of their predicted SNPs, whereas only up to 46 % of the SNPs reported by Samtools are shared by the other methods (Fig. 2d). Whereas NGSEP identifies 80 and 75 % of the SNPs reported by GATK and Tassel respectively, GATK and Tassel respectively identify 62 and 69 % of the SNPs reported by NGSEP. Differences in the SNPs retained by the four methods can occur due to genotype calls confidently predicted by one method and not called by other method that produce changes in the number of individuals genotyped, or due to discrepancies in the genotype calls that produce different estimates of observed heterozygosity. To rule out the latter option, we calculated the percentage of SNPs in the filtered datasets that are contained in the non filtered datasets provided by each method (Fig. 2d) and we found that close to 90 % of the filtered SNPs identified by each method are identified by at least other method. Whereas over 99 % of the SNPs within the Samtools or the GATK filtered datasets appear in the NGSEP non filtered dataset, only 72 and 90 % of the SNPs within the filtered NGSEP dataset appear in the non filtered datasets of GATK and Samtools respectively. Moreover, we verified that more than 96 % of the genotype calls contained by a filtered dataset are consistent with genotype calls predicted by other methods. The largest difference between methods (3.57 %) was observed between the filtered Samtools dataset and the unfiltered Tassel dataset. The percentages of inconsistency between the genotype calls shared between the filtered NGSEP dataset and the unfiltered datasets of GATK, Samtools and Tassel are 2.41 %, 2.77 % and 3.17 % respectively. Over 99 % of these discrepancies are genotypes called homozygous by one method and heterozygous by another method. A similar comparison performed with the datasets obtained from the NxA family shows results consistent with this comparison.

As a starting point for the comparison of different methods, we calculated the number of genotypes and the number of errors as a function of the minimum quality score (GQ format field in the VCF file) because this score should be related to the confidence assigned by each method to each genotype call. We found that quality scores of GATK and Samtools tend to be smaller than those of NGSEP (Additional file 1: Figure S1). On the other hand, most of the quality scores reported by Tassel are higher than those of NGSEP, ranging between 66 and 100.

**Fig. 3:**
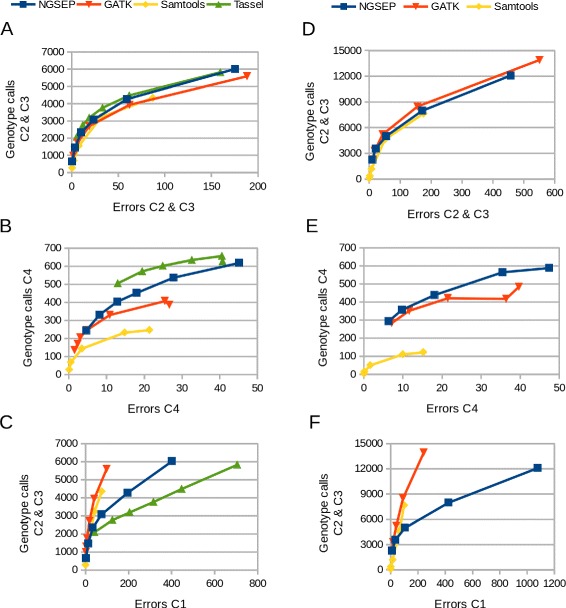
Quality assessment for cassava F1 families. Top figures: Number of genotype calls in SNPs classified in the categories that are useful to build a genetic map (C2 and C3, see Methods for details) contrasted with the number of segregation errors identified in such categories in **a** the K family and **d** the NxA family. Middle figures: Number of genotype calls in SNPs segregating the two parents (C4) contrasted with the number of (false) homozygous genotypes called in SNPs catalogued in this category in **b** the K family and **e** the NxA family. Bottom figures: Number of genotype calls in SNPs classified in the categories C2 and C3 contrasted with the number of genotyping errors identified in SNPs predicted to be monomorphic in **c** the K family and **f** the NxA family. For each pipeline the dots represent datapoints obtained filtering genotype calls at different minimum quality scores. Values in all figures are thousands of genotype calls

Similar to previous benchmarks [17], we contrasted the number of genotype calls in SNPs useful to build a genetic map (categories 2 and 3) against the number of errors detected within these categories. Figure 3a and d show that the four methods provide roughly equivalent high accuracies in this analysis, being NGSEP and Tassel slightly more accurate than GATK and Samtools in the K family and GATK slightly more accurate than NGSEP and Samtools in the NxA family. Samtools is the most conservative algorithm reporting at *q*≤10 about the same number of SNPs than NGSEP at *q*≥60, however with more errors.

Although SNPs in category 4 are in principle not useful for construction of genetic maps from F1 populations, we performed the same analysis for the SNPs in this category to assess the behavior of each method in highly heterozygous sites. In this case Tassel and NGSEP more clearly outperform GATK and Samtools (Fig. 3b and e). We believe that the reason for this outcome is that Samtools and GATK assume that SNPs tend to be in Hardy-Weinberg Equilibrium (HWE) [12], which is not the case for SNPs in categories 2 and 4. For the case of Samtools, the strand bias filter, which we could not deactivate, can also be a reason for reduced sensitivity, taking into account that an important percentage of the genome covered in a GBS experiment is only sequenced in one strand, and only fragments with lengths equal or smaller than the read length are sequenced in the two DNA strands. Tassel up to this point seems to have the best accuracy, although we could not assess this pipeline using the NxA family data. Finally, because we filtered out SNPs monomorphic for the alternative allele, the remaining predicted SNPs with low observed heterozygosity (*H*_*o*_≤0.2) are likely to be called due to genotyping errors. In this comparison Tassel showed the worst behavior generating 28,954 spurious SNPs at *q*≥99, compared to 5,953 spurious SNPs produced by NGSEP at *q*≥40 and about 1,000 produced by GATK and Samtools at *q*≥20 and *q*≥10 respectively. Figure 3c and f show the number of predicted erroneous genotype calls in monomorphic sites contrasted with the number of genotype calls useful to build genetic maps as a measure of sensitivity. NGSEP tends to produce more singleton errors than GATK and Samtools but the assignment of quality scores given by NGSEP seems to be more effective than that given by Tassel to filter out these errors. Overall, these results suggest that Tassel has a tendency to call heterozygous genotypes over homozygous genotypes which makes it appear as having the best accuracy on the highly heterozygous sites at the expense of having the worst accuracy on monomorphic sites. Results obtained with the bean population (next section) seem to confirm this hypothesis.

As a final assessment of the accuracy of the NGSEP pipeline, we built a draft genetic map for the NxA population using as input one of the most high-quality datasets obtained with NGSEP, which includes 2,422 SNPs. Consistent with previous studies [4, 8], we obtained 18 linkage groups, which we could uniquely map to the 18 chromosomes assembled in the latest version of the reference genome available in phytozome [23], having only 27 SNPs inconsistent with the chromosome assignment of each linkage group. Sorting of SNPs within each linkage group largely coincides with their predicted physical positions in the reference genome (Additional file 1: Figure S2). The number of SNPs mapped to each chromosome was on average 142.8, ranging from 62 mapped to chromosome 16 to 359 mapped to chromosome 1. The total length of the map (2,615 cM) was relatively close to the 2,412 cM reported by the International Cassava Genetic Map Consortium (ICGMC) based on the consensus of nine genetic maps [8] and the 2,571cM reported by [4] for the K family. The average intervals between two adjacent mapped markers was 1.33 cM. The largest group, with a total length of 190.15 cM, was mapped to chromosome 1, whereas the smallest group, with 98.75 cM, was mapped to chromosome 17. Chromosome 1 also contains the highest marker density, with an interval average of 0.6 cM, whereas the least saturated group maps to chromosome 16, with an interval average of 1.33 cM. The longest interval between adjacent SNPs is observed on chromosome 13 with a value of 35.174 cM.

### Comparison of methods on a common bean MAGIC population

**Fig. 4:**
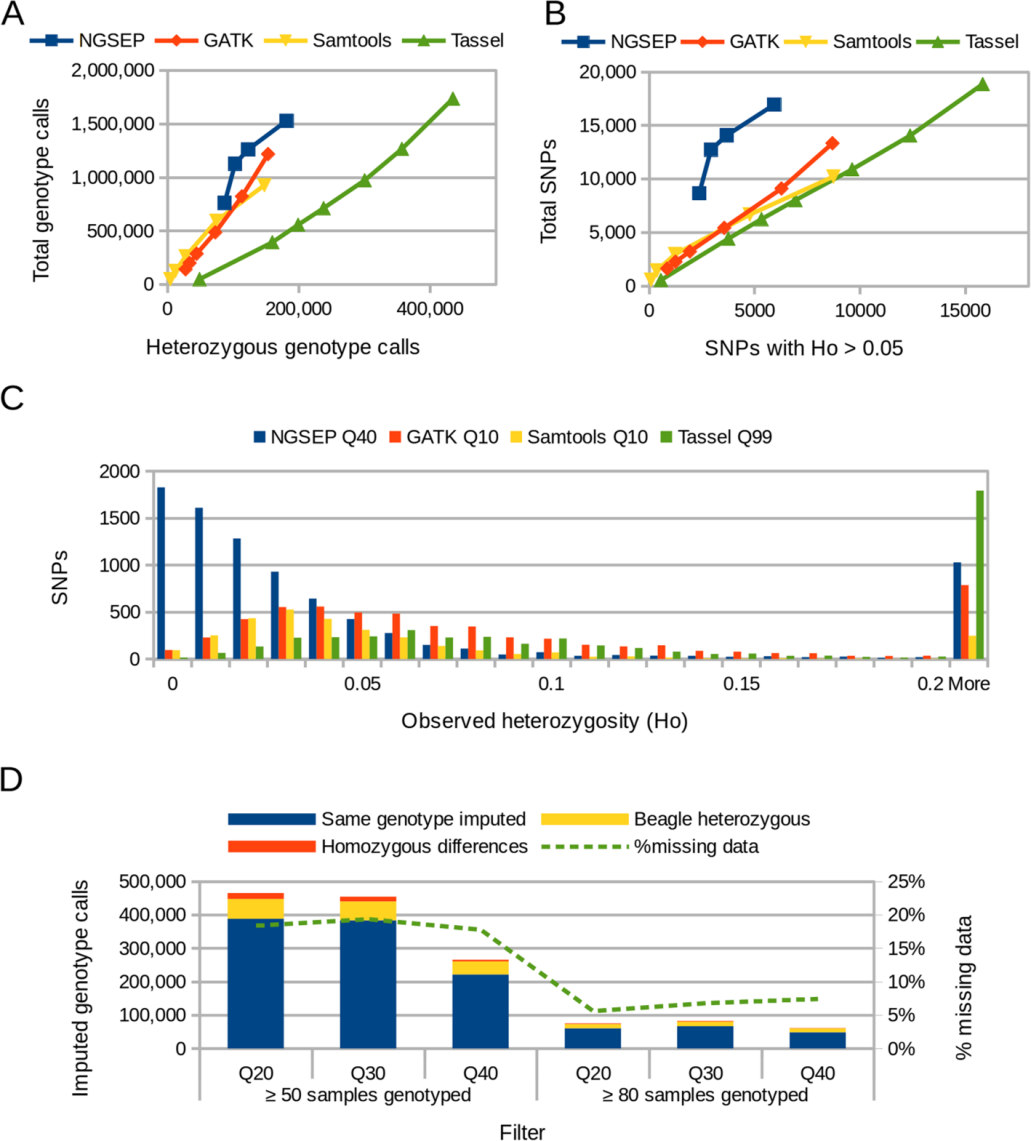
Quality assessment for the bean MAGIC population. **a** Total number of genotype calls obtained from sequencing data for the bean MAGIC population contrasted with the number of heterozygous genotype calls. For each pipeline the dots represent datapoints obtained filtering genotype calls at different minimum quality scores. **b** Total number of SNPs obtained in the same experiments as a function of the number of SNPs with observed heterozygosity larger than 0.05. **c** Distribution of observed heterozygosity for datasets obtained with the four pipelines compared in this study. **d** Distribution of imputed genotype calls for different datasets obtained with NGSEP and imputed with NGSEP and with Beagle. The green line represents the percentage of the total dataset that imputed genotype calls represent for each dataset

As a benchmark for comparison of methods on populations of inbred lines, we present here preliminary results on GBS of a common bean multiparental advanced generation intercross (MAGIC) population [24, 25] developed by the bean program of the International Center for Tropical Agriculture (CIAT, see Methods for details). We analyzed one of the lanes sequenced for this project, including a subset of the population composed by 7 parental lines and 88 siblings. After running the different pipelines and filtering using different quality scores, we removed SNPs in regions masked as repetitive in the bean reference genome [26] as well as variants with less than 80 individuals genotyped and variants with MAF below 0.05. In this case we have more variability in allele frequencies and therefore we can infer less information from the structure of the population. However, assuming that the development of the population achieved random mating of the eight parental lines, we can infer that the percentage of heterozygous genotypes per site ranges from 25 to 50 %. After the four rounds of inbreeding performed to obtain F5 plants the percentage of heterozygosity should range from 1.5 to 3 %. Hence, we assumed for this quality assessment that most of the heterozygous genotype calls predicted by the different pipelines would be errors and we contrasted the total number of genotypes predicted by each method with the number of those that were heterozygous. Figure 4a shows that NGSEP provided the best accuracy on this comparisons whereas Tassel produced the largest number of probably false heterozygous calls.

We verified if heterozygous genotypes are clustered in a few particular SNPs, which could happen due to repeats not identified in the reference genome or even due to balancing selection, or if they are spread over the whole dataset as expected for errors. We contrasted the total number of SNPs obtained by each method against the number of SNPs with observed heterozygosity (*H*_*o*_) larger than 0.05, which in this subset of the population would correspond to SNPs in which at least 5 individuals are heterozygous. Figure 4b shows that NGSEP also achieved the best behavior in this comparison mainly because most of the heterozygous genotype calls predicted by NGSEP are clustered in about 25 % of the SNPs, whereas heterozygous genotype calls predicted by GATK, Samtools and Tassel are spread over more than 50 % of the SNPs. Figure 4c shows that only NGSEP produces an *H*_*o*_ distribution consistent with the expected percentage of heterozygous calls for this population. In the case of Samtools and GATK, the most likely explanation for this behavior is the effect of the assumption of Hardy-Weinberg equilibrium which in this dataset does not hold due to inbreeding. Tassel on the other hand is probably overcalling heterozygous genotypes because, unlike the other pipelines, it does not take into account base quality scores during genotype calling.

Finally, we performed a comparison between the imputation module developed in NGSEP for inbred populations against Beagle [20], which is one of the most widely used tools for genotype imputation and, to the best of our knowledge, the only imputation tool able to process files in VCF format. We considered six different scenarios combining filters on quality and minimum number of individuals. Figure 4d shows that the same calls are predicted by both methods in about 82 % of the imputed genotypes and that between 80 and 90 % of the differences (12 to 20 % of the imputed genotypes) happened because Beagle imputed heterozygous genotypes. Although a better gold-standard dataset and more algorithms should be considered to perform a formal comparison between methods for imputation on inbred populations, this initial result suggests that the HMM implemented in NGSEP can provide genotype calls with accuracies similar to other state-of-the-art software tools for genotype imputation.

## Discussion

Genotype By Sequencing (GBS) is a powerful and cost-effective protocol to assess the variability of entire populations. However, maximizing the information obtained from GBS experiments requires a bioinformatics pipeline able to understand the particular nature of the reads produced by this protocol and adaptable to optimize its behavior on populations with different characteristics and in particular different distributions of variability and heterozygosity. Here we report the novel functionalities of NGSEP that were designed and implemented to facilitate the analysis of GBS data, and we demonstrated that NGSEP is a powerful and adaptable framework to genotype populations of an outcrosser highly heterozygous species such as cassava as well as populations of inbred individuals such as the bean MAGIC population. For the complete MAGIC population, we assembled a highly curated dataset of about 20,000 SNPs which is now being used to perform genetic mapping of complex agronomically relevant traits in bean (Manuscript in preparation). Besides the populations described in this study and recent works on analysis of whole genome sequencing data in rice [27] and peanut [28], the NGSEP pipeline has been used by different collaborators to obtain genomic variation datasets from WGS, GBS and RAD-sequencing data taken from different populations of landraces, breeding materials and even wild relatives of rice, cassava, beans, potato, lettuce and sugar cane.

We compared the accuracy of NGSEP against that of other widely used software packages for analysis of High throughput Sequencing (HTS) data such as GATK and Samtools, which were originally optimized for low coverage WGS data in humans, and the Tassel GBS pipeline which was specifically built for the GBS protocol developed at Cornell. We believe that this effort itself is a novel contribution to the field because, to the best of our knowledge, comparisons of methods for SNP discovery and genotyping using formal benchmark datasets as a baseline are generally missing from current literature, especially for GBS data and non-model organisms. Our comparisons show that NGSEP provides the best overall accuracy under different scenarios.

Comparison against tools commonly used for SNP discovery in human genetics shows that NGSEP outperforms GATK and Samtools mainly because optimization of these tools for high accuracy on low coverage WGS data included the assumption of Hardy-Weinberg Equilibrium (HWE) which does not hold on highly heterozygous sites commonly found on F1 populations, or sites with low heterozygosity such as most of the variable sites in populations of inbred species. Strand bias filters are also not generally adequate for GBS data which probably explains why Samtools reported the lowest sensitivity in these experiments, taking into account that this tool was among the best ranked in our previous benchmarks with WGS data [17].

Compared to Tassel, which is currently the preferred tool for analysis of GBS data, NGSEP also provides better overall accuracy mainly because Tassel seems to overcall heterozygous genotypes. The most likely reason explaining this behavior is that, unlike the other pipelines and for efficiency reasons, Tassel disregards the information included in base quality scores, which restricts to relative allele counts the information to call genotypes. Hence, even sequencing errors tagged during primary analysis with low base quality scores could produce false heterozygous genotypes. We believe that this is the main reason explaining the unexpectedly high number of predicted variants with low MAF in the cassava F1 populations and the large percentage of heterozygous calls in the bean MAGIC population. Other difficulty we found while trying to compare Tassel with other tools was that we could not find a way to run the pipeline on the NxA cassava population because the raw reads were already distributed per sample and were paired-end and not single-end. This issue makes a clear advantage for NGSEP, Samtools, and GATK over Tassel in terms of usability because these pipelines can be directly used on data produced by different GBS protocols.

In our experiments Tassel is the most efficient tool for GBS data analysis, followed by Samtools. However in both cases the improved efficiency comes at the cost of lower accuracy. This difference can be critical in applications such as GWAS because several markers with different allele frequencies need to be accurately genotyped within each genomic region to increase the chance of discovering true correlations between genomic and trait variation.

We are currently carrying on different efforts to improve the usability of NGSEP, to allow more researchers acquire the capacity to analyze HTS data with lower technical support. Among other improvements, we released for our graphical interface a wizard that greatly simplifies the amount of workload needed to obtain datasets of variants from HTS, and in particular GBS data. Most of the functionalities of NGSEP can also be deployed in a command line environment or integrated in a web portal solution such as Galaxy [29]. We are also working with major bioinformatics software integration platforms such as CyVerse [30] to allow more users to benefit from the integrated analysis of entire populations achieved by NGSEP.

## Conclusion

Bioinformatic analysis of GBS data with NGSEP provides a powerful framework for discovery and genotyping of thousands of genetic markers in entire populations. Analysis of several populations of different crops shows that NGSEP currently provides a great balance between completeness, accuracy, efficiency and usability compared to other software packages. We expect to keep expanding NGSEP with novel functionalities to facilitate genetic mapping of complex traits and to assist the genetic improvement of staple crops through the use of molecular breeding techniques such as genomic selection.

## Methods

### Development of NGSEP and parameters used to compare with other tools

NGSEP is written in Java 1.6. It can be deployed as a standalone command-line program, a Graphical User Interface (GUI) implemented as an Eclipse IDE plugin, or using the XML scripts for integration in the Galaxy [29] environment. NGSEP relies on the libraries jsci-core for statistics, sam-1.68 to manipulate sam/bam files, the SortSam package of Picard [31] to sort alignments and Xchart [32] to produce charts in the graphical interface. The GUI includes a screen for read mapping with bowtie2 [11]. NGSEP reads and writes standard formats for genomic analyses, such as BAM, VCF or GFF.

Single-end raw reads from the K family and the MAGIC population were demultiplexed using the Deconvolute module of NGSEP, while paired-end reads from the NxA family were already demultiplexed. Because adaptor contamination was identified in about 35 % of the raw reads sequenced from the K family and the MAGIC population, the first 6-mer of the adaptor sequence (AGATCG) was searched through every read and reads showing this sequenced were trimmed to the initial basepair of this sequence. Trimmed reads were aligned to the Phaseolus vulgaris v1.0 [26] and the Manihot esculenta v4.1 [33] reference genomes, using bowtie2 [11] with default parametes, except for the maximum fragment length of the paired-end reads in the NxA population, which was set to 1,000 bp. Alignments were coordinate-sorted and indexed using Picard. We performed the discovery of variants by running the FindVariants module on all the samples of each population. As required for GBS data, the detection of repetitive regions, copy number variants (CNVs) and other structural variants was turned off; the maximum base quality score was set to 30 and the minimum quality for reporting a variant was set to 40; one base at the 5’ end and five bases at the 3’ end of each read were ignored. Also, due to the nature of GBS experiments, the maximum number of reads allowed to start at the same position was raised to 100. Finally, the prior heterozigozity rate was set to 10^−4^ in the bean population and to 10^−3^ in both cassava populations. The VCF files produced at this step were used as input for the MergeVariants module, to get the list of variant sites of each population. Afterwards, all samples were genotyped at the variant sites by running the FindVariants module again, keeping all parameters unchanged except for the minimum variant quality score, which we set to zero to retain as many genotype calls as possible. Finally, the MergeVCF module was used to join all the VCFs into a single file for each population.

### GATK pipeline

Demultiplexed reads were mapped to their corresponding reference genome using bwa-mem v0.7.12 [10] with the -M flag on, mapped reads were then coordinate-sorted and merged into a single file per population using the MergeSamFiles tool from Picard v1.134. Duplicates were not marked because in GBS samples stacked reads are confused with PCR duplicates and then the MarkDuplicates module filters between 60 and 95 % of the aligned reads. For genotyping of the three populations, bam files were processed using the Genome Analysis Tool Kit (GATK) v3.4.0 [13], following their suggested best practices. For the data cleanup step, the four tools were pipelined: RealignerTargetCreator, IndelRealigner, BaseRecalibrator and PrintReads, using always default parameters. As we did not have a SNP or indel database for cassava or beans, we provided the algorithm with an empty VCF for recalibration. In the case of the NxA population, the Quality Score Base Recalibration could not be performed because of computer memory limitations, and because some samples were sequenced twice (see “Methods” of [8]), producing different sequencer biases. For the variant discovery step, the HaplotypeCaller was used with both standard minimum confidence thresholds (for emitting and calling a variant) set to zero, to allow a higher sensitivity. The prior heterozigozity was set to 10^−4^ in the bean population and to 10^−3^ in both cassava populations. All other parameters were left with default values.

### Samtools pipeline

Alignments from bowtie2 [11] were analyzed using Samtools and BCFtools v1.2 [12]. For this purpose, the mpileup command from Samtools was used over all the samples simultaneously, with default parameters, and the resulting pileup was stored in a BCF file. This file was analyzed with the call command from BCFtools, using the multiallelic caller model, and keeping all possible alternative alleles to achieve maximum sensitivity. The mutation rate was set to 10^−4^ for the bean population, whereas for the cassava populations was left to its default value of 10^−3^.

### Tassel pipeline

The raw reads from the sequencer were analyzed using the GBS pipeline from Tassel 3 [15]. The tools were run consecutively in the following order: the FastqToTagCountPlugin, with the ApeKI enzyme for both, the bean MAGIC and the cassava K family populations. Then, the MergeMultipleTagCountPlugin and the TagCountToFastqPlugin were run with a minimum of three for the times a tag needs to appear to be output. Afterwards, the sequences in fastq format were mapped to each reference genome using bowtie2 in the very sensitive (local) mode, and the alignment was converted to the tagsOnPhysicalMap (.topm) format using the SAMConverterPlugin. Next, the original fastq reads and the tags count were used to obtain the tagsByTaxa files in the tbt.byte format using the FastqToTBTPlugin, and (in the K family) were subsequently merged using the MergeTagsByTaxaFilesPlugin applying the option to identify taxa with identical names. Finally, SNPs were called using default parameters and stored in VCF format using the tbt2vcfPlugin along the whole genome. A custom script was used to concatenate the SNPs called for each scaffold in the reference genome into a single VCF file.

### Development and sequencing of F1 cassava populations

We reanalyzed GBS data taken from two recently sequenced cassava full-sib F1 families. The first family, referred here as the K family, was developed and sequenced as an effort leaded by Universidad Nacional de Colombia to build a highly dense SNP based genetic map useful to identify genes related to resistance to cassava mosaic disease (CMD) and cassava bacterial blight (CBB) [4]. The population, initially developed at CIAT, consists of 137 individuals derived from a cross between cultivars TMS30572 and CM2177-2. GBS was performed at Cornell University to obtain reads from both the parents and their siblings.

The second family, referred here as the NxA family, is the largest of the nine biparental populations developed by the International Cassava Genetic Map Consortium (ICGMC) to assemble into chromosomes the current reference genome [8]. This family, developed at the International Institute for Tropical Agriculture (IITA) and sequenced at the Vincent J. Coates Genomic Sequencing Laboratory (VCGSL), consists of 303 full siblings derived from the cross of the African cultivars Namikonga and Albert.

For both biparental populations, we first checked the integrity of the family to detect and filter unrelated samples using two separate methods: 1) comparing the percentage of homozygous differences between the parental lines and the offspring, and 2) calculating the identity by descent (IBD) coefficient using the software package King [34]. Excluding also samples with low number of sequenced reads (less than 50 Mbp), a total of 119 samples for the K family and 248 samples for the NxA family were finally utilized.

### Comparison of pipelines for SNP detection from GBS data in F1 families

For each family we ran the four SNP calling pipelines described in this study using different values for minimum genotype quality scores. Then we filtered out SNPs that are monomorphic for the alternative allele and SNPs in regions of the reference genome masked as repetitive. Additionally, we filtered out SNPs with less than 100 individuals genotyped in the K family and less than 200 individuals genotyped in the NxA family. We classified the SNPs obtained on each experiment in four possible categories depending on the Mendelian segregation pattern of a typical F1 family based on the four possible genotype combinations of the parents: 1) AAxAA (Monomorphic) 2) AAxAa, 3) AaxAa, and 4) AAxaa. Because the genotypes of the parents could be missing or could contain genotyping errors, we classified each SNP based on its observed heterozygosity (*H*_*o*_) and minor allele frequency (MAF) within the population instead of using the parental genotypes. Assuming an infinite population, perfect Mendelian segregation and perfect genotyping, expected values for these parameters on each category are: 1) *H*_*o*_=0, MAF=0, 2) *H*_*o*_=0.5, MAF=0.25, 3) *H*_*o*_=0.5, MAF=0.5 and 4) *H*_*o*_=1, MAF = 0.5. To account for deviations from these assumptions, we classified the SNPs using these thresholds: 1) *H*_*o*_<0.2, 2) 0.2≤*H*_*o*_≤0.8 and MAF ≤ 0.37, 3) 0.2≤*H*_*o*_≤0.8 and MAF > 0.37, and 4) *H*_*o*_>0.8.

In absence of an explicit gold-standard to measure the sensitivity and specificity of each method, we calculated the number of genotypes and the number of segregation errors on each family taking into account the expected genotypes on each category. SNPs falling in category 1 should be monomorphic and hence any genotype with a minor allele (including heterozygous genotypes) should be an error. On the other hand, SNPs in category 4 should be heterozygous in every sibling. Therefore, heterozygous genotypes in the parents or homozygous genotypes in the siblings should be errors. Categories 2 and 3 include SNPs useful to build a genetic map and for QTL analysis. Hence, our main measure of sensitivity is the number of genotypes obtained in SNPs classified in these two categories. Errors in these categories include deviations from the expected genotypes of the parents and homozygous genotypes for the minor allele in category 2.

### Genetic map construction

Map construction was performed using the Maximum Likelihood mapping algorithm implemented in JoinMap 4.1 using the outbreeding full sib family (CP) population type. The main limitation in the number of SNPs included in this analysis was the capacity of the software that did not allow to analyze more than 3,000 SNPs in a 8GB memory machine. We therefore selected the more useful and high-quality SNPs for the construction of the genetic map. First, from the total 228,187 SNPs generated with the best quality in NGSEP (Q60), we filtered out SNPs in repetitive regions and with less than 96 % of missing data, to obtain 26,146 SNPs. Then, we selected SNPs with an observed heterozygosity (Ho) between 0.4 and 0.6, resulting in 4,327 SNPs. Furthermore, we only uploaded to JoinMap the 3,869 SNPs having informative parental genotypes and consistent segregation patterns. After the conventional filters applied in JoinMap (goodness of fit and redundancy of data) we were able to unambiguously map 2,422 SNPs. A LOD threshold of 5.5 was used to construct 18 linkage groups, corresponding to the 18 cassava chromosomes. We used a two point grouping analysis to assign markers to their corresponding linkage group, and within groups they were mapped using the strongest crosslink (SCL). Also, the map was generated using a recombination frequency below 0.45.

To validate the accuracy of the genetic map, scaffolds from the version 4.1 of the cassava reference genome were aligned to the recently released version 6.1, available at phytozome [23] using blast searches. To obtain the most likely origin of each scaffold, only hits with lengths at least 50 % of the length of the best hit, mapping to chromosomes, and with identity score higher than 90 % were retained. Remaining segments of scaffolds aligning to multiple sites were also removed. The most likely chromosomal position of each SNP was calculated translating the coordinate within the scaffold where the SNP was discovered to chromosomal coordinates, using the alignment of the scaffold segment closest to the SNP in scaffold coordinates.

### Development and sequencing of a bean MAGIC population

A common bean Multi Parental Advanced Generation Inter Cross population (MAGIC) was developed from 8 parental CIAT breeding lines (SXB 412, INB 827, ALB 213, SEN 56, SRC 2, MIB 778, SCR 9, INB 841), selected to combine desirable characteristics such as tolerance to heat stress, yield under drought and growth under unfavorable soil conditions such as high Aluminum or low Phosphorous stress. Genotypes were combined by pairwise crosses and subsequently crosses of F1 plants. After the F2 population lines were advanced by single seed descent to F5, when DNA was extracted from young leaves (Additional file 1: Figure S3). We performed GBS on 648 F5:7 plants plus the 8 parental lines following the Cornell protocol [7] and using NGSEP for bioinformatic analysis. Total genomic DNA was extracted from 25-days-old seedlings plants. 1 gr of leaf tissue was frozen using liquid nitrogen and ground to a fine powder with metal balls in a paint shaker for three minutes. The DNA was extracted with the Urea based DNA extraction midi prep protocol [35]. The quantity of the DNA was measured with NanoDrop 1000 and the DNA integrity was visualized trough EcoRI, PstI and RsaI restriction enzyme digestion in a 1 % agarose gel electrophoresis. DNA was sent to the Cornell sequencing facility, where GBS libraries were generated based on the Cornell protocol [7] using ApeKI as restriction enzyme. Sequencing was carried out at the Cornell sequencing facility on the Illumina HiSeq platform. A subset of 95 MAGIC lines is used here as a real example data set to compare NGSEP to other GBS data analysis tools.

## Abbreviations

BAM, binary sequence alignment/map format; BCF, binary variant call format; CBB, cassava bacterial blight; CIAT, International Center for tropical agriculture; CMD, cassava mosaic disease; CNV, copy number variant; DNA, deoxyribonucleic acid; GATK, The genome analysis toolkit; GBS, genotype by sequencing; GFF, general feature format; GPL, general public license; GQ, genotype quality; GUI, graphical user interface; GWAS, genome-wide association studies; HMM, Hidden Markov model; HTS, high throughput sequencing; HWE, Hardy-Weinberg equilibrium; IBD, identity by descent; ICGMC, International cassava genetic map consortium; IDE, integrated development environment; IITA, International institute for tropical agriculture; LD, linkage disequilibrium; LOD, logarithm of the odds; MAF, minor allele frequency; MAGIC, multiparental advanced generation intercross; NGSEP, next generation sequencing eclipse plugin; PCR, polymerase chain reaction; QTL, quantitative trait locus; RAD, restriction site associated DNA; SAM, sequence alignment/map format; SCL, strongest crosslink; SNP, single nucleotide polymorfism; VCF, variant call format; VCGLS, Vincent J. Coates genomic sequencing laboratory; WGS, whole genome sequencing; XML, extensible markup language

## Additional file

Additional file 1 Supplementary figures referred in this manuscript. (PDF 90 kb)
