# Supplementary material for: Bioinformatic analysis of genotype by sequencing (GBS) data with NGSEP
Source: BMC Genomics. 2016 Aug 31;17(Suppl 5):498. doi: 10.1186/s12864-016-2827-7 (PMC5009557; doi:10.1186/s12864-016-2827-7)

**Supplementary figure 1**. Number of genotype calls and number of errors for different quality scores on the datasets obtained from the analysis of the cassava K family population. All values in the Y axis are thousands of genotype calls

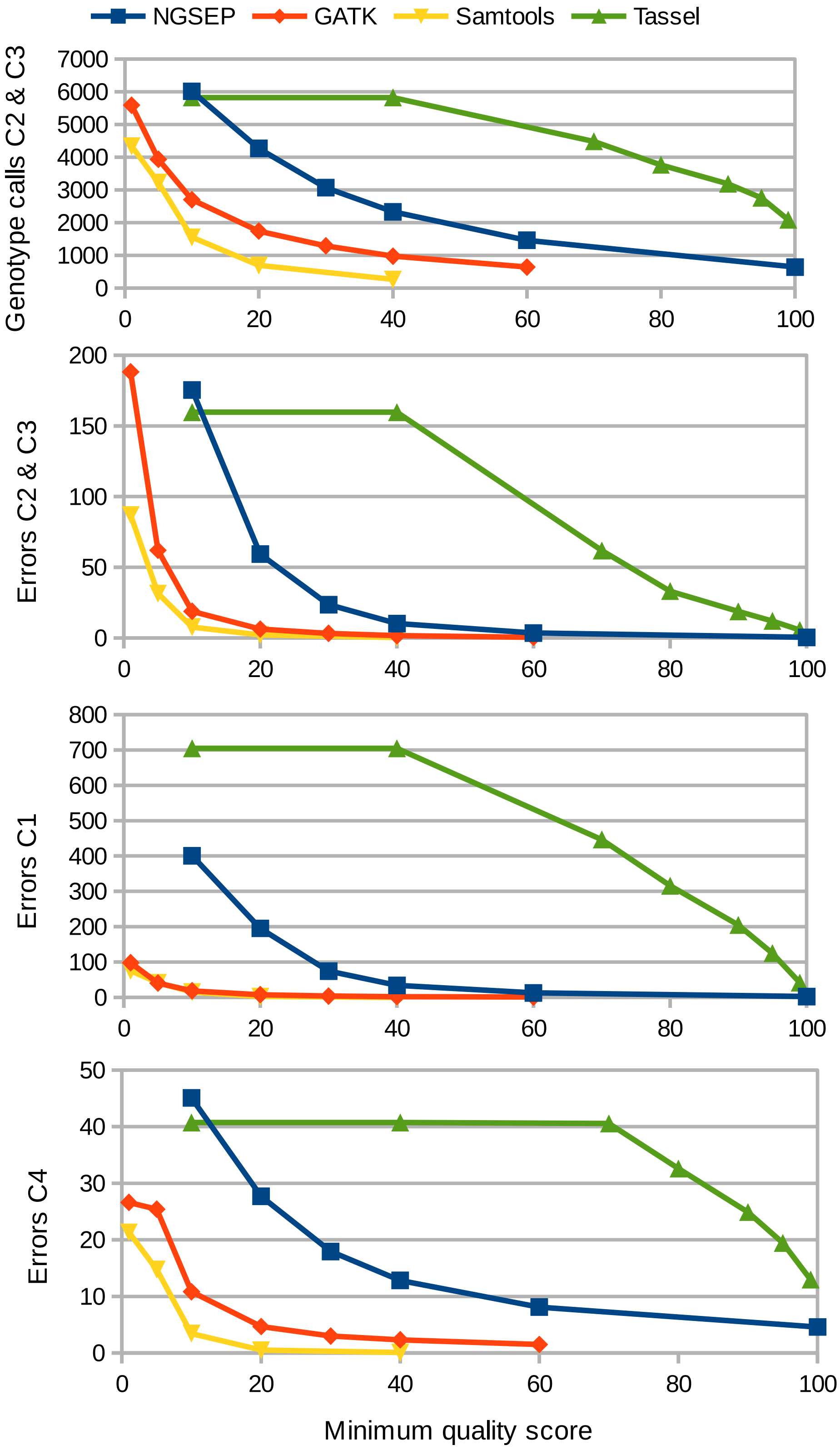

**Supplementary figure 2**. Dot plot of predicted genomic sites in the latest cassava reference genome (v6.1) for the SNPs organized in the 18 linkage groups obtained after the construction of a draft genetic map using the genotype calls predicted by NGSEP on the cassava NxA population

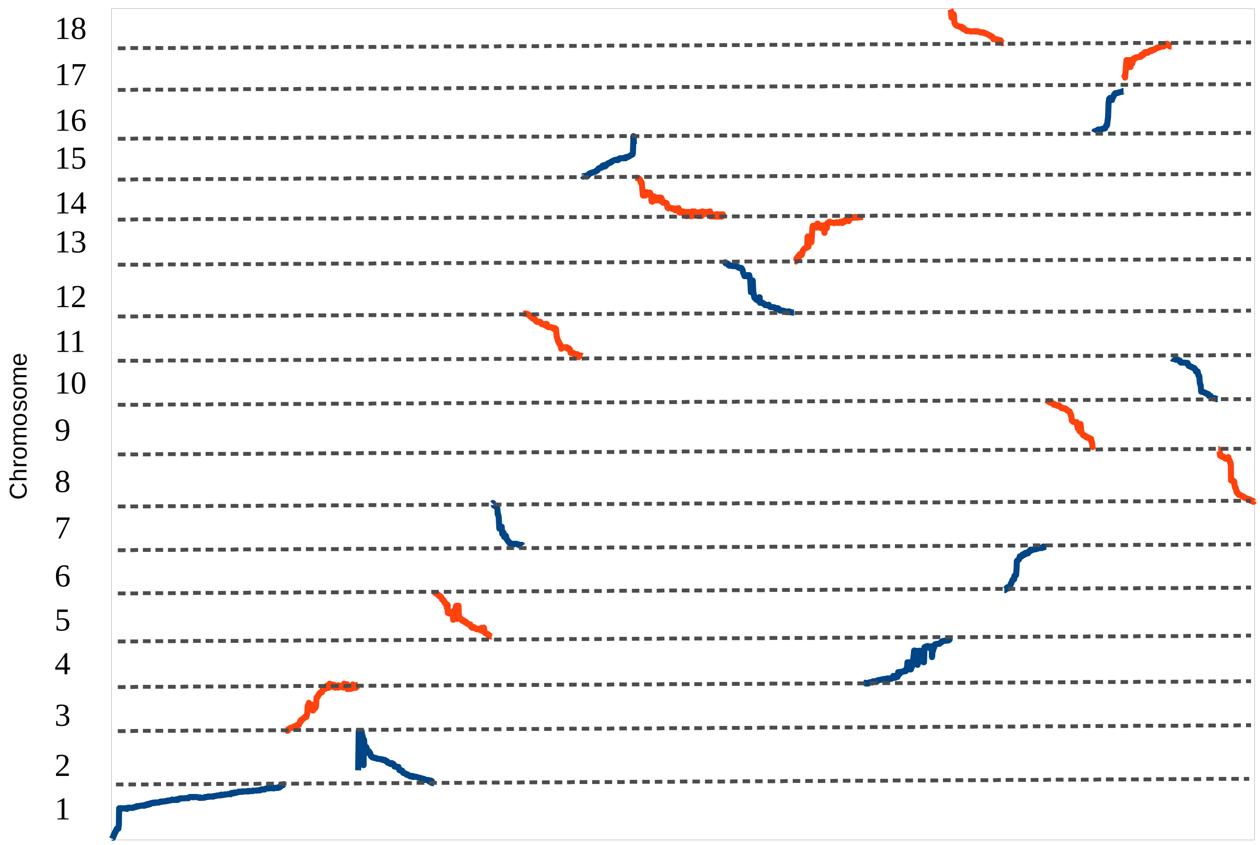

**Supplementary figure 3**. Schematic of the development of the common bean MAGIC population

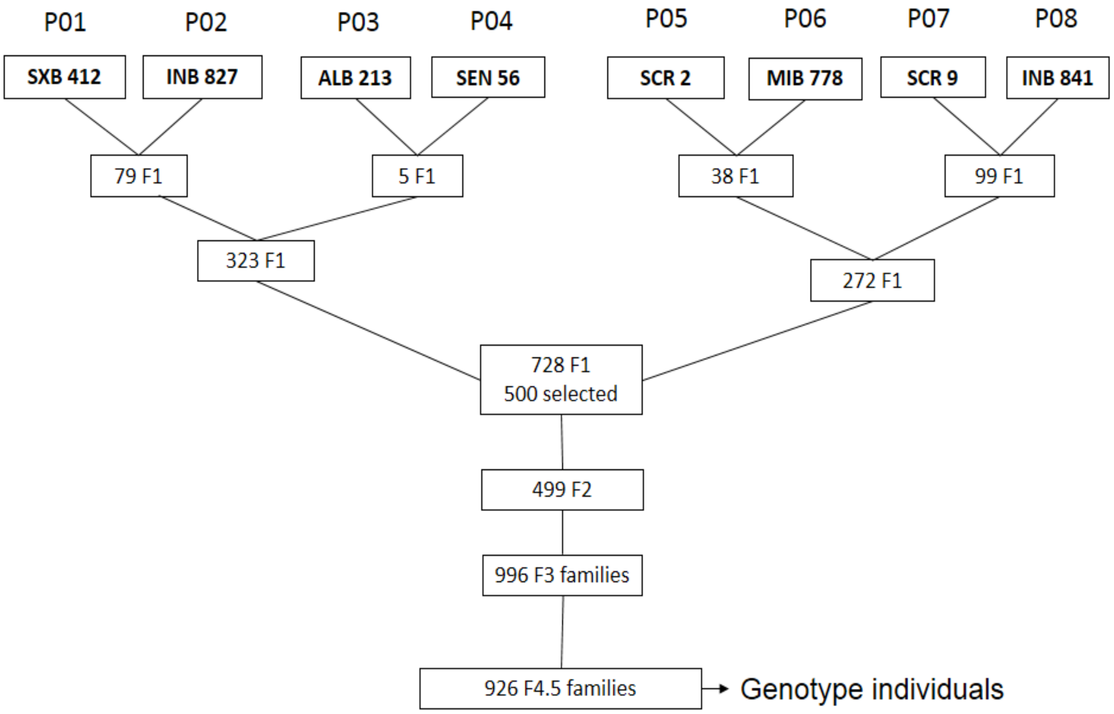

Supplement: Additional file 1 — Supplementary figures referred in this manuscript. (PDF 90 kb) [file 12864_2016_2827_MOESM1_ESM.pdf]
